# Machine learning approaches for data-driven hydrocarbon bioaugmentation and phytoremediation: the role of multi-omics insights

**DOI:** 10.3389/fmicb.2026.1742848

**Published:** 2026-03-05

**Authors:** Ugochukwu Chukwuma Okafor, Saeed M. Alghamdi, Lorna Anguilano, Yang Yang

**Affiliations:** 1 Department of Applied Microbiology and Brewing, Faculty of Biosciences, Nnamdi Azikiwe University, Awka, Nigeria; 2 Respiratory Care Program, Clinical Technology Department, Faculty of Applied Medical Sciences, Umm Al-Qura University, Makkah, Saudi Arabia; 3 Experimental Techniques Centre, Brunel University London, Uxbridge, United Kingdom; 4 Department of Chemical Engineering, Brunel University London, Uxbridge, United Kingdom

**Keywords:** bio-augmentation, cancer-risk mitigation, hydrocarbon contamination, machine learning, multi-omics, phytoremediation

## Abstract

Hydrocarbon contamination, particularly with polycyclic aromatic hydrocarbons (PAHs), poses a significant environmental challenge due to its persistence and carcinogenic effects on ecosystems and human health globally. This review explores how ML algorithms can enhance the efficiency of bio-augmentation and phytoremediation through predictive modeling, real-time optimization of microbial consortia, and plant species selection. Traditional bioremediation methods, such as bioaugmentation and phytoremediation, are characterized by slow degradation rates and sub-optimal performance in complex, multi-contaminant environmental milieus. The use of machine learning (ML) models with multi-omics data presents an advanced predictive approach to optimizing bioremediation processes by providing a systematic understanding of microbial and plant-mediated hydrocarbon degradation strategies and processes. ML models can predict which microbial strains or plant species will effectively degrade hydrocarbons under specific environmental conditions by utilizing supervised learning methods such as support vector machines and neural networks. Additionally, the combination of multi-omics data with ML facilitates the identification of critical genes, enzymes, and metabolic pathways involved in the degradation of hydrocarbons, and offers insights into the molecular mechanisms which drive the bioremediation process. The translation of laboratory-based ML models into large-scale, real-world bioremediation strategy is hindered by the complex, dynamic nature of our contaminated environments. This review paper showcases these hinderances and provides a direction for future research, including the development of field-deployable technologies, adaptive ML models, and real-time environmental monitoring strategies. The integration of ML with multi-omics holds substantial promise for enhanced efficiency, adaptability, and scalability of bioremediation strategies which ultimately mitigates carcinogenic risks often associated with hydrocarbon-polluted lithosphere.

## 1 Introduction

Soil contamination by hydrocarbons including polycyclic aromatic hydrocarbons (PAHs), aliphatic hydrocarbons, and volatile organic compounds (VOCs), constitutes a major environmental challenge due to their persistence, toxicity, and mobility in terrestrial ecosystems: according to Correia and Rasteiro (2025), hydrocarbon pollutants alter soil physicochemical properties, reduce nutrient availability, and degrade ecosystem productivity. PAHs and VOCs, in particular, are bioaccumulative and carcinogenic, posing serious threats to human health through inhalation, ingestion, or dermal exposure, also Enuneku et al. (2020) emphasize that in regions near industrial sites, oil wells, or refineries, local populations face chronic exposure that may lead to respiratory problems, neurological disorders, and increased cancer risk. The carcinogenic potential of hydrocarbons, particularly PAHs, is extensively documented in toxicological and epidemiological research: according to Jameson (2019), several PAHs including benzo[a]pyrene are classified as Group 1 carcinogens by the IARC, with strong associations to lung, skin, bladder, and gastrointestinal cancer, similarly, Shimada and Fujii-Kuriyama (2004) emphasized that once metabolized by enzymes such as cytochrome P450, PAHs generate reactive degradation products capable of directly altering DNA, also, Williams et al. (2020) reported increased cancer incidence in populations residing near industrial zones and oil refineries, attributing it largely to chronic PAH exposure.

Bioremediation techniques like bio-augmentation and phytoremediation have so far served as potential solutions for hydrocarbon contamination management (Kakde and Sharma, 2024; Okafor et al., 2016a), similarly, Paniagua-Michel and Fathepure (2018) highlighted the potential of microbial consortia in increasing the degradation rate of petroleum hydrocarbons in contaminated environments where-in the added microbes often possess catabolic genes that enable them to metabolize complex hydrocarbons into less toxic compounds. Bio-augmentation entails the application of specialized microbial consortia to degrade hydrocarbons through enzymatic breakdown, while phytoremediation entails the use of plants to extract, degrade, or detoxify pollutants (Okafor et al., 2016a) and according to Okafor et al. (2016b), bioaugmentation with hydrocarbon-degrading bacteria significantly improved the bioremediation of oil-contaminated soil, especially when native microbial populations were insufficient. Despite their promising potentials, both processes are faced with several limitations, which includes the inefficiency of microbial degradation pathways under fluctuating environmental conditions, the slow growth process and slow pollutant uptake rates of plants, and most strikingly, the complex interactions between the soil, pollutants, and the bioremediators themselves (Liu P. et al., 2025), and also, Liu P. et al. (2025) noted that the survival and activity of introduced microbes are often compromised *in situ*, limiting their ability to degrade hydrocarbons efficiently over time. Consequently, these traditional remediation methods most often have limitations in addressing the degree of contamination and the long-term ecological and health hazards posed by the hydrocarbon contaminants, additionally, Ambaye et al. (2022) emphasized that complex hydrocarbon mixtures, especially in aged or weathered contamination, often resist biological degradation, necessitating supplemental or alternative remediation strategies.

Recent advancements in machine learning (ML) have introduced innovative strategies to optimize bioremediation processes through the enabling of predictive modeling, real-time adjustments to varying environmental conditions, as well as personalized strategies for both microbial and plant selection (Blessing and Olateru, 2025) and demonstrated that ML models could accurately predict hydrocarbon degradation rates under varying conditions, reducing the reliance on trial-and-error methods in field applications. Machine learning, particularly when integrated with multi-omics data, has demonstrated great potential for the enhancement of the efficacy of bio-augmentation and phytoremediation through well-targeted design of microbial consortia, plant-mediated pollutant uptake optimization, as well as real-time monitoring of soil health (Ayub et al., 2025), also, Contreras-Salgado et al. (2024) emphasized that ML and systems biology tools can be used to rationally design synthetic microbial communities tailored for bioremediation tasks. Machine learning algorithms can determine and process a wide array of datasets from environmental monitoring tools, genomic sequencing, and remote sensing strategies to predict the most effective remediation methods, enhancing both the speed and accuracy in mitigating hydrocarbon contamination toxicity (Li et al., 2025), similarly, Prabu et al. (2025) emphasized that ML techniques, when applied to integrated datasets, outperform traditional models in predicting contaminant behavior and optimizing cleanup strategies. Zamfir et al. (2025) also noted that the use of machine learning models for integrating large-scale environmental and biological datasets can improve the selection and performance of remediation strategies and methods.

For hydrocarbon-contaminated soils, ML-optimized bio-augmentation and phytoremediation represent a paradigm shift, first in enhancing pollutant degradation and also in addressing the public health implications associated with carcinogenic hydrocarbon exposures (Das et al., 2025; Tesfaye et al., 2025) reported that ML-optimized microbial communities enhanced the degradation of PAHs by over 60% compared to conventional methods. These advanced technologies hold wider promise for ecological restoration, thus providing scalable solutions for the reclamation of polluted lithosphere and the restoration of ecosystem services, like biodiversity and soil fertility (Nassary, 2025; Okafor, 2023),

Multi-omics refers to the examination of levels of various biological information that interact within an organism or ecosystem simultaneously and multi-omics data including genomics, transcriptomics, proteomics, and metabolomics can be utilized by researchers to have an understanding of how microbes and plants operate to degrade contaminants (Hayes et al., 2024). Multi-omics data refers to lots of different types of data together; which involves mixing machine learning with genomics, transcriptomics, proteomics as well as metabolomics.

This review aims to critically assess the potential of machine learning algorithms in optimizing bio-augmentation and phytoremediation for hydrocarbon-contaminated soils, enhance the predictability of pollutant degradation, and how multi-omics insights provide data-driven solutions for mitigating carcinogenic risks in polluted soils.

## 2 Hydrocarbon contamination and carcinogenic risks

Hydrocarbon pollutants primarily originating from petroleum extraction, refining, and accidental spills are recognized as among the most persistent and ecotoxic compounds in both terrestrial and aquatic environments, their environmental stability and bioaccumulative nature make them long-term hazards: according to Paniagua-Michel and Fathepure (2018) and Patel et al. (2020), petroleum hydrocarbons such as PAHs are not only toxic to microbial and aquatic life but also alter soil properties and suppress native microbial communities. Also, Bejarano and Michel (2016) emphasized that oil spills have catastrophic effects on marine biodiversity, particularly in coastal ecosystems, by smothering organisms and disrupting trophic interactions.

The toxicity of hydrocarbons is strongly influenced by their chemical structure, with PAHs recognized as particularly hazardous due to their carcinogenic and mutagenic potential: according to Jameson (2019), several PAHs: such as benzo[a]pyrene, are classified as Group 1 human carcinogens due to their proven role in DNA adduct formation and genetic mutations, furthermore, Bukowska et al. (2022) highlight that PAHs can cross the placental barrier, posing risks to fetal development. Despite regulatory frameworks, the complexity and variety of PAHs present a challenge in establishing comprehensive exposure thresholds and ensuring public safety.

Central sources such as industrial emissions, oil spills, and contaminated soils release hydrocarbons into the environment. Arrows indicate exposure routes leading to human health impacts (carcinogenicity, respiratory, neurological, cardiovascular, and developmental effects) and environmental impacts (soil degradation, aquatic toxicity, atmospheric greenhouse gas emissions, and crop contamination). Environmental degradation further amplifies human exposure, highlighting the interconnected nature of these risks.

**Figure 1:**
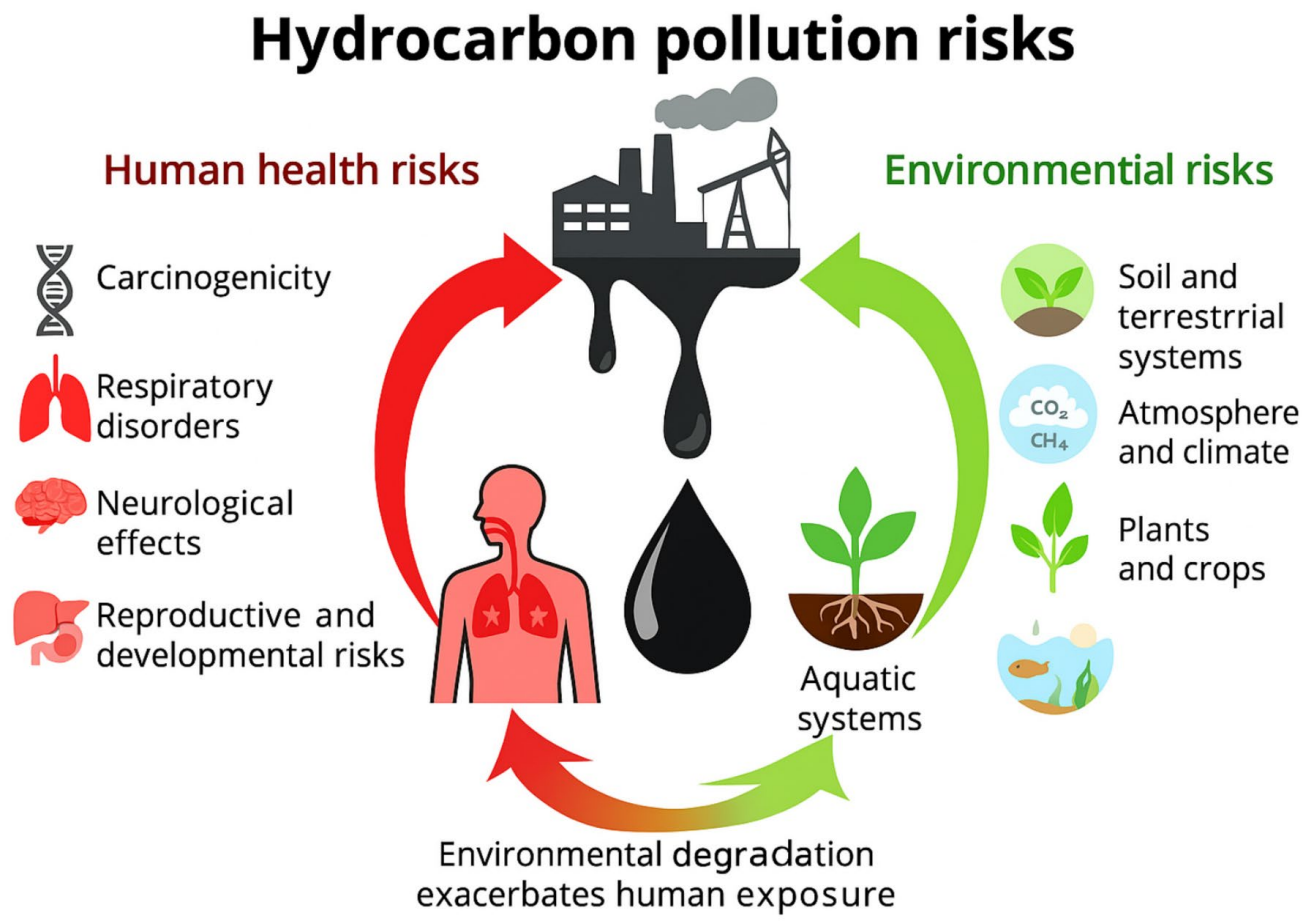
Schematic representation of hydrocarbon pollution pathways and associated risks, based on Yang et al. (2017).

Figure 1 summarizes the levels of hazards posed to humans and environments by PAH and Hydrocarbons, indicating their multi-layer effects.

Box 1 **Risks to human health and ecosystems due to polycyclic aromatic hydrocarbons (PAHs).**

PAHs are derived from crude oil and are described as having 2 or more aromatic rings (Carey, 2018). Due to their ability to form fused aromatic rings, PAHs are chemically stable and hydrophobic. This leads to PAHs to being resistant to degradation. This causes PAHs to persist in the environment, where they can bioaccumulate and be transported over large ecological distances.

PAHs like benzo[a]pyrene, benz[a]anthracene, and dibenzo[a,h]anthracene have been linked to the development of cancer through their ability to form DNA adducts (Haritash and Kaushik, 2009). The International Agency for Research on Cancer classifies these 3 PAHs as group 1 human carcinogens. They have been linked to the development of liver, lung, and skin cancer, and they can also cross the placenta, causing developmental and reproductive toxicity.

PAHs also have the ability to disrupt entire ecosystems. They can reduce the activity of microorganisms, and disrupt the cycling of nutrients. Nutrient uptake and photosynthesis are inhibited, and in terrestrial ecosystems, PAHs can disrupt trophic levels through bioaccumulation in soil fauna. The disruption of food webs and bioaccumulation of PAHs can also be observed in coastal and marine ecosystems. These also face the additional threat of being smothered by oil. This can reduce the overall biodiversity of these ecosystems (Padilla-Garfias et al., 2024).

Challenges in defining comprehensive regulatory thresholds for PAHs continue to persist due to their chemical heterogeneity and the different manners in which people are exposed to PAHs (skin contact, breathing, and through what they eat) (Prabu et al., 2025). As such, the need for efficient strategies to remediate PAHs in the environment is clear.

### 2.1 Sources and environmental distribution of hydrocarbons

Hydrocarbons contaminate soils as a result of the various activities involved in the petroleum industry and the subsequent consequences of the industry, including petroleum extraction, refining, transportation, industrial discharge, and the processes of incomplete combustion (Kianfar, 2025; Sui et al., 2021). From all of the contaminants, PAHs are the most concerning because they are very persistent, hydrophobic, and have a high tendency to accumulate in the environment, including soils and sediments (Srivastava et al., 2019; Adipah, 2018). Hydrocarbons have the potential to migrate and undergo different processes, including adsorption to soil organic matter and leaching to ground water, which transform the hydrocarbons into long-term contaminants of the environment (Ahmed et al., 2022).

Box 1 summarizes the most important toxicological and ecological consequences of PAHs and their potential impact.

### 2.2 Health risks of hydrocarbons

Dermal contact with hydrocarbons in contaminated soils, inhalation of volatilized and/or evaporated hydrocarbons, and consumption of contaminated food and water are the pathways through which humans come into contact with petroleum hydrocarbons (Abdulai et al., 2025; Mallah et al., 2022). Based on toxicological and epidemiological studies, chronic exposure to PAHs, and in particular, high molecular weight PAHs such as benzo[a]pyrene, have been shown to have carcinogenic, mutagenic, and teratogenic effects. Because of the potential risk to human health, several PAHs have been designated as priority pollutants by various international regulatory bodies (Mallah et al., 2022).

Box 1 presents a summary of the health risks and exposure pathways of PAHs.

### 2.3 Effects of soil and water contaminated with hydrocarbons

The potential presence of some hydrocarbons could adversely affect the health of all organisms on the planet, but the presence of hydrocarbons in the soil and water systems negatively impacts the ecosystems in a more profound way (Cavazzoli et al., 2022; Sutormin et al., 2024). Hydrocarbons in the soil negatively affect the composition of the different types of microbial communities, decrease the amounts of certain enzymes, and negatively affect the cycling of various nutrients in soil. These deficiencies decrease the resilience of the soil ecosystem (Fei-Baffoe et al., 2024). Adverse effects of hydrocarbons in freshwater systems include negative effects on the organisms that live in the bottom of water bodies, the accumulation of toxic substances in organisms at various levels of the food chain, and the reduction of the number of different species of organisms in the ecosystem (Xu et al., 2018). These negative ecological impacts make the process of removing the contaminants more difficult, especially in ecosystems that lack nutrients and in systems that contain a lot of different types of soil and sediments (Ite et al., 2018).

Box 1 describes the ecological impacts of PAHs in more detail.

### 2.4 Emerging needs for regulatory frameworks and remediation

Environmental regulation and risk assessment for PAHs are difficult primarily because of the persistence, toxicity, and complexity of exposure pathways associated with them (Sankar et al., 2023). Despite the fact that guideline values have been developed for a few individual compounds, the great variability with soil type, climate, and the level of biological activity in a given area greatly restricts the application of such values (Barbosa et al., 2023; Ram, 2026). Therefore, the need for more flexible, more targeted on-site approaches for remediation focused on overcoming complex mixtures of contaminants has increased.

Box 1 contextualizes the relevance of PAH contamination to public health and regulations.

## 3 Traditional bioremediation techniques: bioaugmentation and phytoremediation

Bioremediation is one of the ways of cleaning soils polluted with hydrocarbons since it employs living organisms, especially microorganisms and plants, to destroy or contain the pollutants (Bala et al., 2022). Among various methods, bioaugmentation and phytoremediation seem to be the most researched ones. As effective as these techniques are in some cases, their capability to solve the challenges posed by the long-lasting nature of hydrocarbon pollutants is limited. This section analyzes these techniques by looking into the principles of bio-augmentation and phytoremediation while assessing their effectiveness and identifying ways to improve them for hydrocarbon degradation (Bala et al., 2022).

### 3.1 Bio-augmentation: microbial enhancement for hydrocarbon degradation

Bio-augmentation is the application of specific microbial strains or consortia to bio-fouled environments in order to enhance the existing microbial degradation of hydrocarbons. In bioaugmentation, and as a consequence of bio-inoculation, the primary bacteria involved will be those which take hydrocarbons as carbon sources or secrete enzymes that degrade hydrocarbon molecules. The principles of bio-augmentation are becoming popular for the reclamation of soil polluted hydrocarbons because of the biodegradation potentials of microorganisms (Medaura et al., 2021).

Hydrocarbon degrading microorganisms have been classified into bacteria, fungi and algae and a few of them are *Pseudomonas, Alcanivorax* and *Mycobacterium* known for their efficient degradation of hydrocarbons (Chakraborty et al., 2025). The bacterial species have special enzymes such as oxidases that are involved in degrading (oxidizing) aliphatic and aromatic hydrocarbons (aminases) (Demanèche et al., 2004). For instance, PAHs are degraded by the Pseudomonas strains via the aromatic-ring cleavage pathway and likewise Oil-pollutants (Ops) are considered (Walton and Buchan, 2023).

Nevertheless, there are a number of limitations to bioaugmentation; despite all the optimistic findings, the introduced microbes do not always survive the harshness of the contaminated depths, including indigenous microbes (Kurniawan et al., 2022). The survival of introduced microbes in the contaminated environment is one of the most significant challenges, also, environmental stressors such as nutrient deficiencies, unfavorable pH levels and high pollutant concentrations can impede microbial growth and activity (Iqbal et al., 2023); Moreover, the introduced microbes often compete with indigenous microbial populations, which can limit their efficacy and persistence. Therefore, the success of bio-augmentation is not solely dependent on the presence of capable microbes but also on creating an environment conducive to their growth and activity (Iqbal et al., 2023).

Another drawback of bio-augmentation is the specificity of the microbial strains exactly tailored to certain hydrocarbons; mixtures of hydrocarbons encountered in real-life contamination situations may contain a multitude of compounds and therefore need to be degraded using consortia of microorganisms (Zhong et al., 2020). This diversity creates the difficulty of choosing the right strains which can co-habit and work together in a way that improves the entire degradation process.

Bioaugmentation can be applied as an effective method for hydrocarbon remediation, however, it compels the use of tailored microbiomes and controlled environments in order to achieve the highest output performance and maximum degradation. Engineered or naturally enriched microbial groups may be custom-made in order to have Catabolic pathways for the degradation of recalcitrant PAHs and long-chain alkanes as hydrocarbons (Zhong et al., 2020; Nwankwegu et al., 2022).

### 3.2 Phytoremediation: removal of hydrocarbon pollutants through plant based methods

Phytoremediation is defined as the use of green plants to remove, degrade or contain environmental pollution like hydrocarbons and this approach harnesses the ability of plants to absorb and metabolically alter pollutants through phytodegradation, phytoextraction, and phytostabilization (Aryal, 2024). Plants have the distinctive potential to absorb hydrocarbons through roots and transform them into less toxic metabolites or sequestering within their tissues thus diminishing the levels and concentrations of contaminants in soil. Phytodegradation is a term used for the process through which plants degrade pollutants either directly within their tissues or through enzymatic reactions facilitated by microorganisms in the rhizosphere (Kafle et al., 2022); and in the case of hydrocarbons, plants such as poplar trees and sunflowers have shown the ability to take up and break down aliphatic and aromatic hydrocarbons in the soil (Alaidaroos, 2023). The initial and main processes that focus on the intake and degradation of hydrocarbons in plants include the uptake of hydrophobic substances through roots followed by transport to the aerial parts where they are transformed through plant enzymatic or microbial action (Pawlik et al., 2017). These transformations may, in some cases, lessen the toxicity of certain compounds.

Phytoremediation is also used in conjunction with phytoextraction, where certain contaminants like hydrocarbons are taken up and stored in plant tissues especially in the leaves and stems where they could be harvested and disposed of Aryal (2024). However, this method is less hydrocarbon centric as large hydrophobic molecules are difficult to translocate through the plant vascular system; Salix (willow) species demonstrate strong phytoextraction capabilities, particularly for low molecular weight hydrocarbons (Urošević et al., 2024).

Phytoremediation offers potential benefits, but also faces challenges. The most prominent challenge is the low rate of hydrocarbon degradation due to plant influence in comparison to microbial aid. Responsiveness differs due to the growth rate of the plant alongside pollutant uptake capacity which limits the address of large-scale contamination and rapid response (Siddique et al., 2024). Further, the efficiency of phytoremediation is highly dependent on the plant species selected, as well as soil conditions such as pH, moisture, as well as the availability of nutrients. *Helianthus annuus* (sunflower) and *Populus* spp. (poplar) have demonstrated efficacy in many soil types; however may not perform well under all environmental conditions (Liu H. et al., 2025).

Additional drawback of phytoremediation is the potential for the contaminants to be returned back into the environment via volatilization or leaching processes from the tissues of plants, but this however poses specific problems for volatile compounds and those compounds not easily metabolized by such plants (Mocek-Płóciniak et al., 2023). Also, hydrocarbons’ clean-up by plants sometimes results in partial immobilization and has the potential for re-contamination in case of the death of the plants or poor harvest operations (Oyedeji et al., 2022).

### 3.3 Synergistic potential and the need for optimization

Relation to technology and the effects of bio-augmentation and phytoremediation show that the latter works in specific cases. Both bio-augmentation and phytoremediation have inherent limitations which hinders their widespread application. Recent optimization efforts therefore focuses on integrating the two methods or employing novel technological innovations. One example of this is phytoaugmentation where bio-augmentation is incorporated to phytoremediation by adding microbes which degrade hydrocarbons into plant roots (Rim Hassen, 2023). As in many cases, the introduction of these microorganisms speeds up the degradation process since roots can be used by microbes and thus improves overall remediation efficiency (Sharma et al., 2022).

Through continued monitoring of ecosystems and applying ML to bio-augmentation, optimization can be achieved to the techniques of bio-augmentation and phytoremediation, for example ML could be applied to predict the best suited consortia or plant species to be used on specific hydrocarbons or to create the best environmental parameters that will maximize degradation efficiency (Sharma et al., 2024). Moreover, the incorporation of Machine Learning into these strategies stands to resolve many of the conventional issues that protein bio-augmentation faces or poor ‘slow’ degradation rates in phytoremediation.

## 4 Machine learning in bio-augmentation optimization

Machine learning has shown significant promise in enhancing the performance of bio-augmentation strategies. ML models, such as deep neural networks (DNNs), decision trees, and support vector machines (SVMs), can predict microbial performance, community dynamics, and pollutant degradation rates (Hernández Medina et al., 2022) (Table 1).

**Table 1:** ML algorithms used in bioremediation and their application scope.

ML algorithm	Type	Common use in bioremediation	Example application	References
Random forest	Supervised	Feature importance, classification	Predicting degradation potential of isolates	Cappelli et al. (2024)
Support vector machine	Supervised	Classification, regression	Soil contamination classification	Liu et al. (2016)
K-Means	Unsupervised	Clustering microbial functions	Grouping functional gene profiles	Lu et al. (2004)
XGBoost	Supervised	High-accuracy modeling	Predicting phytoremediation efficiency	Tarwidi et al. (2023)
Neural networks	Deep learning	High-dimensional data modeling	Time-series prediction of degradation curves	Fang and Cai (2025)
Autoencoders	Deep learning	Dimensionality reduction	Noise filtering in omics data	Martorell-Marugán et al. (2019)

**Figure 2:**
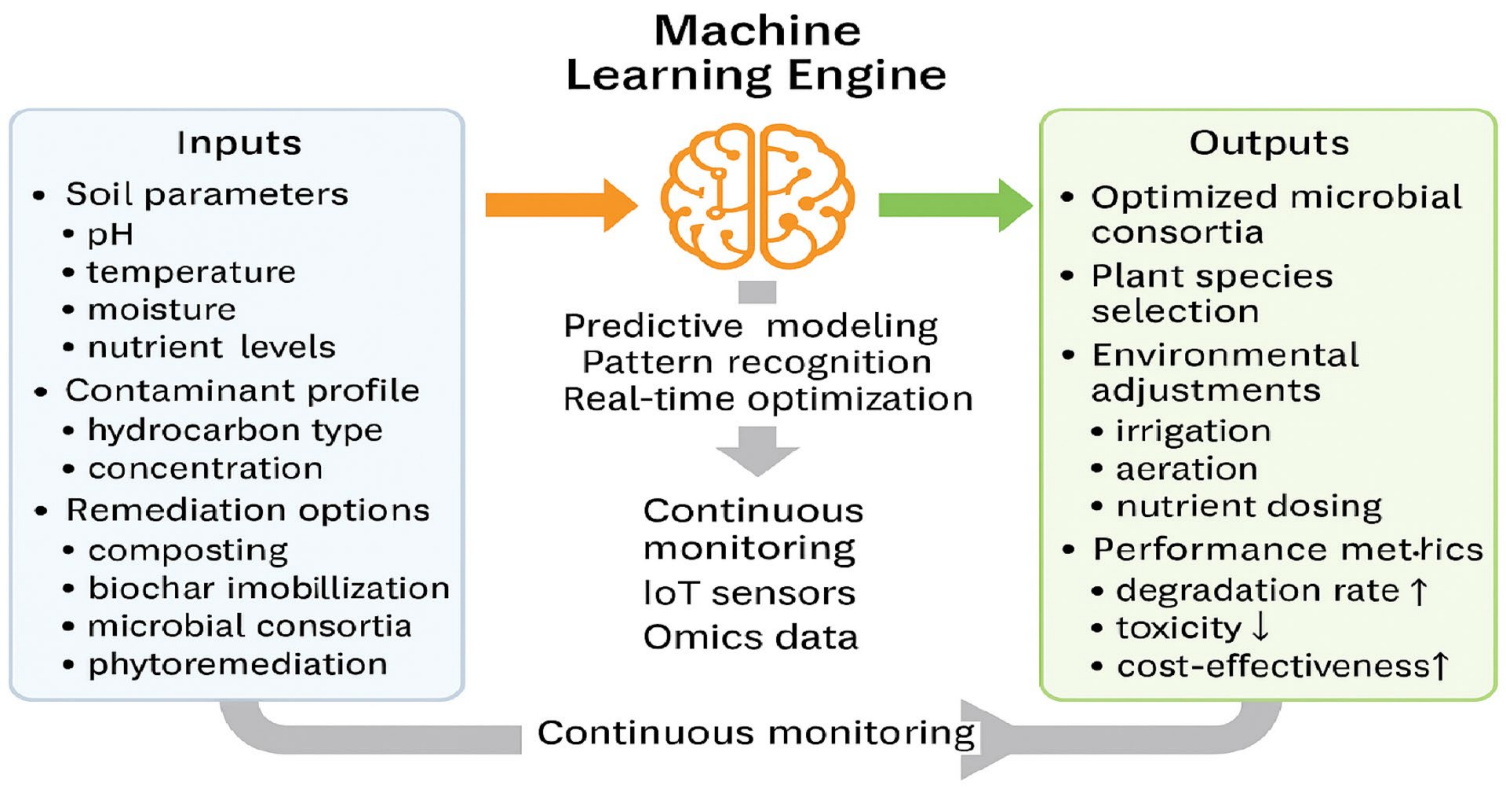
AI-driven optimization of soil bioremediation, based on Wang et al. (2024).

The process of bio-augmentation is the introduction of microbes to accelerate the degrading of pollutants and it is a remediation strategy that is, however, faced with several difficulties in practical scenarios such as microbial survival, environmental adaptability, and the optimization of pollutant degradation rates (Nzila et al., 2016). Traditional methods are often dependent on the empiricism of the chosen microbial strains and the environmental conditions that are deemed optimal for degradation, which leads to inefficient results due to the contamination of complex and dynamic environments (Volkoff et al., 2022). The partnership of machine learning (ML) and bioremediation should definitely be brought up since ML is regarded as a valuable means remediating the above-mentioned obstacles. It is able to give a hand in predictive modeling, real-time optimization, and the increased efficacy of the bioremediation through the use of hydrocarbons in polluted soils (Wu and Zhao, 2023) (Figure 2).

Machine learning models integrate soil parameters, contaminant profiles, and remediation options to predict optimal strategies. Outputs include tailored microbial consortia, plant species selection, and environmental adjustments. Continuous monitoring provides feedback for adaptive optimization, enhancing degradation efficiency and reducing ecological and health risks.

The above figure illustrates the process of soil bioremediation using AI to predict and interpret optimal conditions; including the use of composting, biochar immobilization, as well as other remediation parameters.

Machine learning models, particularly those based on supervised and unsupervised learning, can improve the performance of bioaugmentation by predicting microbial activity, optimizing strain consortia, and fine-tuning environmental conditions to maximize degradation efficiency (Silva-Andrade et al., 2025). The adoption of ML in bioaugmentation enables a more data-driven approach, where microbial consortia are selected based on not only their intrinsic capabilities but also their interactions with one another and the surrounding environment (Wani et al., 2022).

### 4.1 Predictive modeling of microbial degradation pathways

Predicting how tiny (microbial) life forms break down certain paths is an important part of teaching machines how to get better at improving soil health and removing harmful fuel wastes. Techniques like Supervised learning (SL) models that guide machines such as Support Vector Machines (SVMs), Random Forests (RF) and Artificial Neural Networks (ANNs) are quite useful (Qu et al., 2019). These techniques assist in comprehending detailed links between different microbial species and the environmental aspects that positively affect breaking down effectiveness. These techniques work on vast and multi-layered databases. These databases combine the tiny creatures’ properties, like what they are made up of and their enzyme profiles, with how effective they are in breaking down harmful fuel wastes. Secondly, they use the same information with environmental parameters, for example - temperature, pH level (how sour or basic it is), moisture content and oxygen supplies (Minkiewicz et al., 2016).

Recently, scientists have started to appreciate that machine learning (ML) systems (using high-quality integrated genomic and natural data) can efficiently forecast complex breakdown abilities of these tiny lifeforms. This discovery points to a more direct method in soil improvement. Also, these technologies can find important genetic markers and metabolic paths tied to breaking down harmful oils. This means scientists can make informed guesses about these forms’ metabolic capabilities without useless lab testing (Quazi, 2022).

A great example of this approach is the study conducted by Faisal et al. (2025). They successfully used an SVM model to predict how bacterial isolates might break down oil waste using genomic data. They included aspects like the presence or absence of certain genes connected to fuel waste breakdown. This led to the accurate prediction of biodegradation possibilities, showing that SL can make traditional strain selection better, replace labor-intensive and usually inefficient hit-and-miss methods with data-driven candidate prioritization.

### 4.2 Microbial consortia optimization

On analysis of oils that are commonly found in the damaged soils, numerous varieties of compounds are present in those soils. Because of this complexity, using a single strain for restoration work is less effective than using a consortium (or mix) of microbes. ML (Machine Learning) systems can also be used in these situations to design the best arrangement of microbial consortia to be used to improve the rate of degradation (Stepanova et al., 2022). There are also some unsupervised learning methods like clustering and dimensionality reduction, which analyzes datasets that are cumbersome so as to detect microbes that are in consortium via varying metabolic functions (Asnicar et al., 2023).

As an illustration, Patel et al. (2020) conducted a study where they employed a k-means clustering method to group certain microbes which had similar PAHs degradation pathways. This enabled the formation of a microbial consortium which could degrade a larger array of hydrocarbons. This method permits the systematic identification of microbial species which function in synergy, increasing the degradation efficiency microbial and guarantee thorough contaminant breakdown.

In order to adapt to the possible changes of an external environment, the microbial consortia can be reshaped through reinforcement learning techniques. Thus, real-time monitoring via the degradation of process sensors and the adjustment of microbial inoculations continuously form a feedback loop.

### 4.3 Real-time environmental optimization

For bioaugmentation, the environmental conditions such as temperature, pH, moisture and nutrient levels are the influential factors for the microbial activities and pollutant breakdown rates. In the traditional method, we perform a great deal of trial-and-error experimentation in the process of environmental optimization, yet with a lower efficiency and longer time (Muter, 2023). Nevertheless, ML algorithms, particularly those trained in reinforcement learning and predictive analytics, creates the opportunity to optimize environmental conditions in real time (Cipriano et al., 2025).

Reinforcement learning, a domain of ML can incrementally control the environmental constraints in an autonomous fashion and monitor quality dimensions on an ongoing basis to optimize microbial activity. In reinforcement learning frameworks, a program may change how microbes manage moisture and soil nutrients (Treloar et al., 2020).

## 5 Data integration and multi-omics approaches in bioremediation

During bioremediation, one of the new inroads is the use of multi-omics data (lots of different types of data together); which involves mixing machine learning with genomics, transcriptomics, proteomics as well as metabolomics. This high-profile combination gives deeper understanding of how hydrocarbonoclastic microbes break down hydrocarbons. Integrating machine learning with genomic data and metabolic pathways gives better predictions. More appropriate pathways and the most suitable microbes for transforming specific hydrocarbons might be set off in certain environments by so doing (Mukherjee et al., 2017).

In traditional bioremediation, the use of simplistic or incomplete data influences the clean-up process depending upon contaminant concentration, microbial community structure and more. Yet hydrocarbon breakdown involves complex and dynamic interactions between microorganisms, plants and numerous environmental factors (Kakde and Sharma, 2024) (Table 2).

**Table 2:** Multi-omics applications in hydrocarbon bioremediation.

Omics type	Biological insight provided	Example tools/technologies	ML application	References
Genomics	Hydrocarbon degradation genes	Illumina, PacBio, Nanopore	Gene annotation, pathway prediction	Anuraj Nayarisseri et al. (2025)
Transcriptomics	Gene expression under pollutant stress	RNA-seq	Time-series modeling, clustering	Kumarathilaka et al. (2021)
Proteomics	Identification of functional enzymes	LC–MS/MS, 2D-GE	Functional annotation, protein classification	Bharagava and Saxena (2020)
Metabolomics	Metabolic pathway flux under pollutant pressure	GC–MS, NMR	Pattern recognition, metabolite correlation	Wishart et al. (2022)
Microbiomics	Shifts in microbial community and consortia dynamics	16S rRNA, metagenomics	Community profiling, taxonomy prediction	Hidalgo et al. (2020)

In this section, the role of multi-omics data and ML integration in improving soil bioremediation is discussed with regards to: (i) microbial community dynamics, (ii) surveying pollutant degradation pathways, and (iii) real-time optimization of the environment.

With the use of high-throughput technologies to generate additional large amounts of data and through the application of MLs to identify useful patterns, predictive models can be made to streamline and direct bioremediation processes of various hydrocarbons.

**Figure 3:**
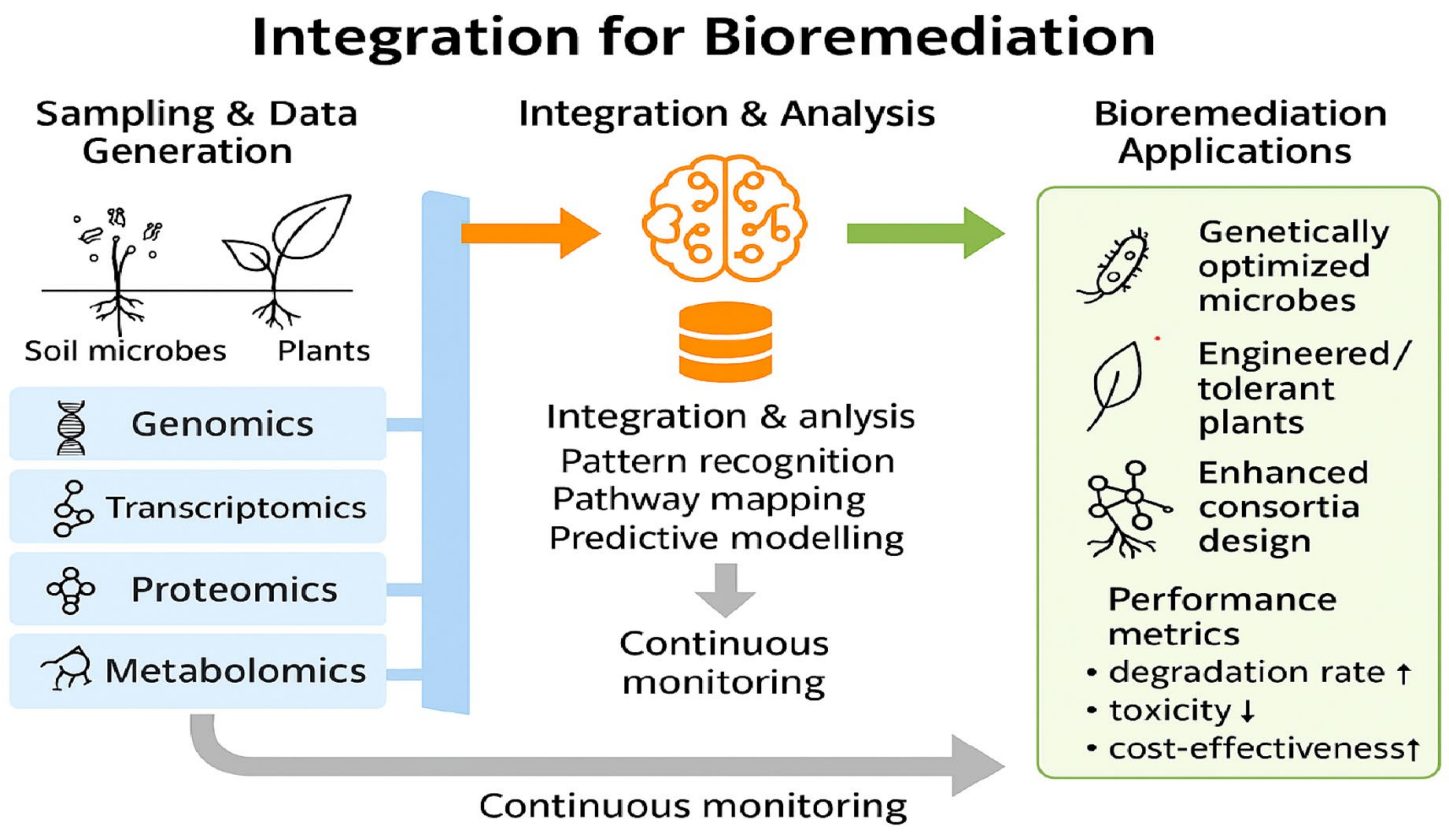
Integration of multi-omics data with machine learning for bioremediation (Verma et al., 2021).

Figure 3 shows how omics technologies can be used in the process of bioremediation. Soil microbes and plants provide genomic, transcriptomic, proteomic, and metabolomic datasets. These are integrated and analyzed by AI/ML models to identify key pathways, predict degradation performance, and design optimized microbial consortia and plant systems. Outputs include enhanced pollutant degradation, reduced toxicity, and improved ecosystem recovery, supported by continuous monitoring and feedback.

### 5.1 Multi-omics data in soil bioremediation

Multi-omics refers to the examination of levels of various biological information that interact within an organism or ecosystem simultaneously and multi-omics data including genomics, transcriptomics, proteomics, and metabolomics can be utilized by researchers to have an understanding of how microbes and plants operate to degrade contaminants (Gutierrez Reyes et al., 2024); also, Machine learning (ML) provides a strong approach to the analysis of multi-omics data, and this analysis allows identifying the main genes, pathways, and metabolic networks involved in the hydrocarbon uptake, tolerance, and degradation (Godoy et al., 2024). Research by Raza et al. (2024) demonstrated that ML models used transcriptomic and metabolomic data to identify stress-responsive genes of plants exposed to petroleum hydrocarbons, as such, these requirements could identify tolerant genotypes.

According to research by Sharma et al. (2020), machine learning can promote the identification of interactions among plant and microbial omics, allowing for the construction of optimized plant-microbe consortia with improved remediation potentials; In addition, deep learning algorithms have been employed to link multi-omics profiles with environmental performance metrics, providing accurate predictions of phytoremediation outcomes, even in complex contamination scenarios (Mohamedikbal et al., 2025).

Machine learning utilizes models like neural networks and random forests to examine the relationships between multi-omics data, predicts the metabolic pathways activated by pollutant degradation, as well as the impact of environmental factors. Machine learning also enhances the degradation efficiency of plants through these advances in technology and consequently enhances phytoremediation (Sartori et al., 2025).

Machine learning has brought transformative ways of interpreting intricate multi-omics data in bioremediation studies; also, through the identification of occult patterns in such large datasets, ML enables us to anticipate the performance of microbial populations and indicates which omics features are truly important in degrading contaminants (Arjmand et al., 2022).

An excellent illustration of this is a study by Fenibo et al. (2025), in which they applied ML to both metagenomic and metabolomic data to predict how effectively various soil microbes would remediate petroleum contamination.

**Figure 4:**
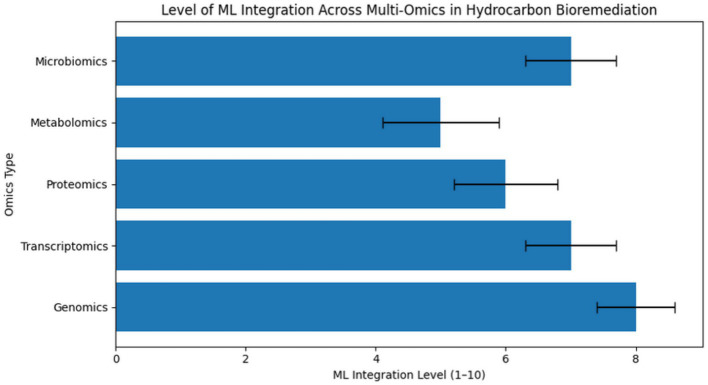
The level of integration of ML with multi-omics for hydrocarbon bioremediation (Reel et al., 2021).

As observed in Figure 4, researchers are combining various types of omics data with ML methods in hydrocarbon bioremediation research.

### 5.2 Machine learning for predictive modeling of bioremediation

Machine learning is changing the way we clean up environmental messes, especially when it comes to oil spills and other organic pollutants; as such there are many variables that can affect the cleanup process, like the chemistry of the soil and how microbes interact with each other (Janga et al., 2023). That is where the applicability of ML comes in. ML can find patterns as well as connections in huge amounts of varying data that people might miss.

Researchers have been very successful at applying various ML methods such as support vector machines, random forests, and neural networks to determine how pollutants degrade in soil. For instance, Nayarisseri et al. (2025) applied support vector machines to forecast which bacteria would be most effective at degrading hydrocarbons based solely on their genetic sequence. Likewise, Mahalle et al. (2025) created models that were able to predict how effectively microbes would remediate contamination based on soil conditions and the composition of microbes present. These tools are enabling environmental remediation to become more targeted and effective than ever before.

### 5.3 Real-time monitoring and adaptive bioremediation

Adaptive management and real-time monitoring are crucial elements of bioremediation in unpredictable environmental conditions. Results are hampered by conventional methods’ frequent lack of adaptability in dealing with field conditions (temperature, pH, oxygen, and contaminant concentration) that change frequently. Machine learning and real-time monitoring technology enable adaptive bioremediation systems to quickly and efficiently adjust to environmental changes (Akintola, 2024). Important environmental factors and Internet of Things (IoT) devices can be monitored by sensor networks, which can then transmit the data for analysis. ML algorithms will process the data in real-time to find patterns, irregularities, and performance problems. Microbes are stimulated more to break down contaminants more effectively when automated decision-making is used to change moisture levels, add nutrients, or adjust aeration (Katie, 2024).

For instance, artificial neural networks (ANNs) and decision trees have been used to predict the impact of environmental conditions on bioremediation efficiency, leading to controlled field operations (Ibrahim et al., 2024). By managing in advance, delays in remediation are eliminated, costs cut, and reliability enhanced.

## 6 Machine learning in phytoremediation optimization

Phytoremediation can clean the soils filled with hydrocarbon. But, it does not always work perfectly. The lack of rapid absorption of these pollutants, sensitivity of certain species to environment disturbance, and specific limiting of some plants could explain its efficiency (Yan et al., 2020).

ML can determine how effective phytoremediation is, based on soil, plants and contaminant factors. Certainly, there is evidence for this. To illustrate, Rasti et al. (2024) applied some ML algorithms to different plant species and their hydrocarbon degrading ability under different environmental conditions to help researchers select appropriate plant species. Rasti et al. (2024) used ANNs to model the uptake of petroleum hydrocarbons by *Vetiveria zizanioides*, allowing them to save a lot of trial-and-error testing.

By using ML, the temperature, pH, moisture, etc., can be fine-tuned to improve the uptake of pollutants. Through receiving the data on these factors from the sensors, ML models can recommend changes such as irrigation, nutrient delivery (Adamo et al., 2025).

**Figure 5:**
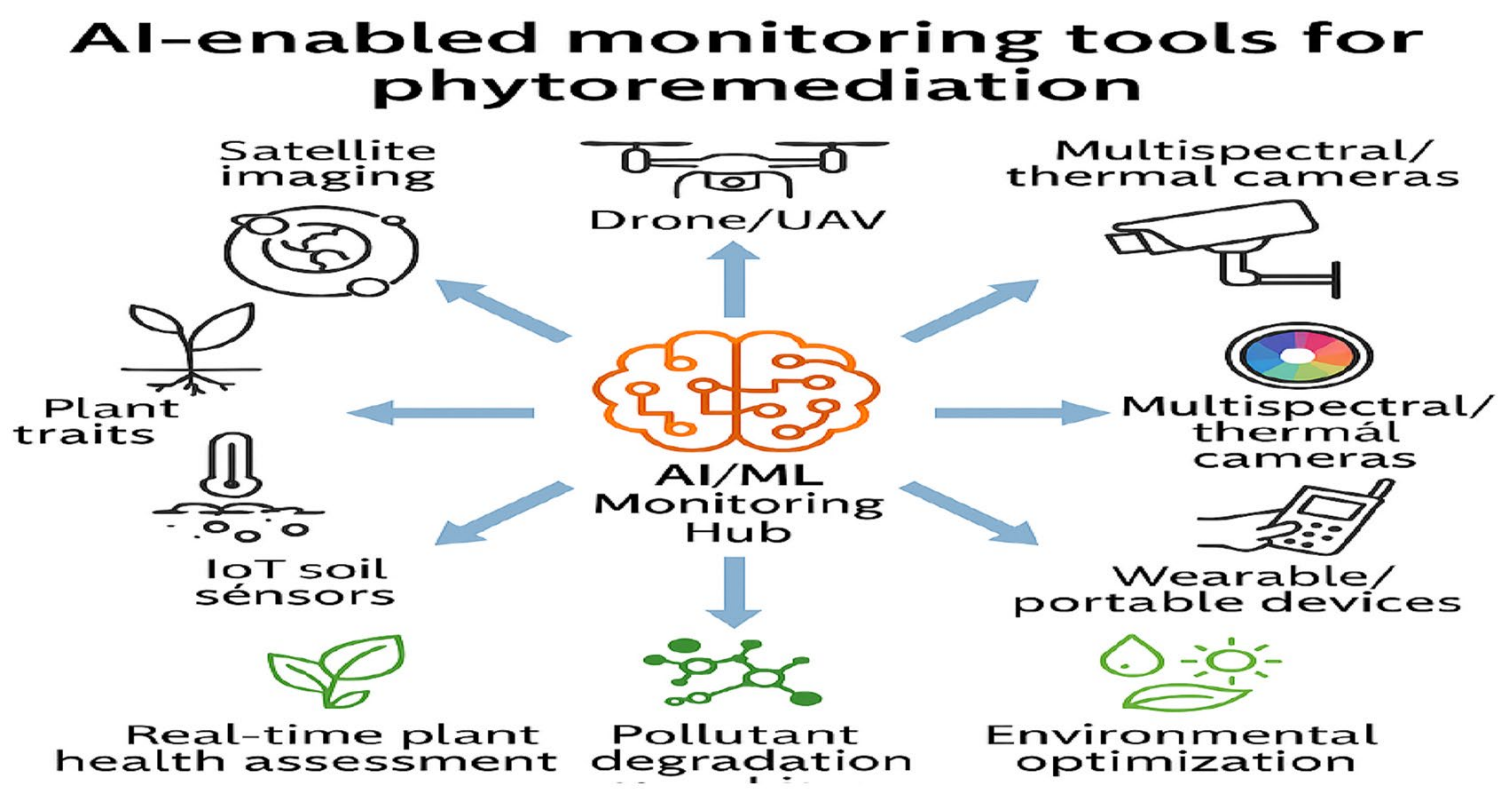
AI-enabled monitoring technologies for phytoremediation (Singh et al., 2022).

Figure 5 illustrates the application of Artificial Intelligence in the field of phytoremediation. These data are explained by utilizing the latest statistical and machine learning tools in MATLAB and Python, including regression models and deep learning approach.

Satellite imaging, drones, fixed and multispectral cameras, IoT soil sensors, and portable devices provide real-time data streams to an AI/ML hub. These inputs are integrated to assess plant health, track pollutant degradation, and optimize environmental conditions for effective phytoremediation.

### 6.1 Predictive modeling of plant-microbe interactions in hydrocarbon degradation

The interactions between plants and soil microorganisms in the rhizosphere play a crucial role in the efficiency of phytoremediation, particularly in the degradation of hydrocarbons (Agarwal et al., 2020). ML models can be applied to predict and optimize these interactions, enabling the selection of plant species that not only enhance pollutant uptake but also stimulate microbial communities capable of degrading hydrocarbons (Tan et al., 2023).

Plant-microbial interactions are the essence of microbial hydrocarbon degradation in phytoremediation. Microorganisms in the rhizosphere facilitate the bioavailability of the contaminant and develop enzymes breaking the hydrocarbons down, and the plants provide root exudates supporting microbial growth (Conlon et al., 2025). Nevertheless, the variability and complexity in the interactions with the quality of soil and contaminants make the interactions difficult to optimize through classical methods.

Machine learning (ML) provides the effective paradigm for describing and forecasting plant-microbe interactions during the breakdown of hydrocarbon contaminants. With large datasets of microbial community signatures, plant characteristics, and environmental parameters, ML discerns tendencies and foretells the optimum plant-microbe pairings for targeted contaminants (Wijaya et al., 2023). Thus, Das and Chandran (2011) predicted the potential for bacterial strains in degrading hydrocarbon contaminants by means of SVM on the basis of the genomic markers. Such models minimize the requirement for expensive lab work and facilitate selective bioaugmentation.

In addition, microorganisms’ community response mechanism in a dynamic root–soil profile, and how microbial consortia interact with plant root system to affect the degradation rate under different soil dynamic conditions have been modeled with ML techniques such as Random Forests and neural networks (Tariq et al., 2025). Incorporation of metagenomic data improves the predictive capability and the control of the microbial inoculants, such as the complementary metabolic pathways (Xamxidin et al., 2025). Existing supervised learning methods such as support vector machines (SVM), random forests, and deep neural networks, may be used to predict how various plant species and microbial consortia interact in soils contaminated with different contaminants (Pace et al., 2025).

Such models can assimilate data emerging from both rhizoremediation and from microbial consortia, hydrocarbon levels, plant growth stage and microbial abundance, in order to identify plant-microbe synergies that increase degradation rates. One example is the study by Deng et al. (2024) which applied an SVM model to predict the potential effect of *Wumerella psychotryphanolytica* and *Ustilago* sp., on rhizospheric microbial community that contribute to improving the degradation of crude oil hydrocarbons within the rhizosphere. By predicting these interactions, ML allows us to identify optimal plant-microbe combinations, which enhances the efficacy of phytoremediation.

### 6.2 Optimization of plant species selection for hydrocarbon uptake

Phytoremediation largely depends on the capability of plants in absorbing, translocating and removing hydrocarbons in contaminated soils. All plants, however, do not have the same potential of uptake and metabolism of the hydrocarbons in the environment. The choice of species of plants suitable in the definition of those with high phytoremediation potential necessitates adequate knowledge on the biochemistry and physiology of the plants that facilitate the absorption and processing of hydrocarbons (Wang and Delavar, 2024).

To be effective in the phytoremediation of hydrocarbon-contaminated soils, an optimization in the choice of plant species is imperative since plant characteristics have a significant role to play in the rate of uptake, root-microbe symbiotic association and the resistance of plants to oil contamination (Zhakypbek et al., 2024). Yet, the conventional techniques of selection are lengthy, and they do not always take into account the interaction of the multi-faceted relationship of plant physiology, type of contaminant and environmental conditions. Machine Learning offers a dependable, data-hungry platform for choosing a suitable plant species through the analysis of large datasets which include soil characteristics, pollutant profiles, plant physiological traits, as well as environmental conditions. Machine Learning models aids in identifying the most efficient plant-pollutant pairings (Niazian and Niedbała, 2020).

### 6.3 Environmental optimization and growth condition adjustment

To illustrate, Bhoi et al. (2021) proved how, through ML models and the coordinated actions of Internet of Things (IoT) devices, one can measure soil parameters and automatically control the irrigation and supply of nutrients, which has a positive impact on the health of plants and the level of remediation.

Besides, sensitivity analysis using ML enables determining the most significant environmental variables influence phytoremediation efficiency to intervene in them (Thoma et al., 2003). This minimizes wastages of input and maximizes the destruction of pollutants particularly in the heterogeneous soils, or marginal soils.

### 6.4 Phytoremediation in complex polluted environments: multi-omics integration

The use of phytoremediation in the environment that has complex pollution (various contaminants are present and interacting with a variety of soil and microbial constituents) needs sophisticated methods to disentangle the various biological processes underlying their removal. Conventional methods tend to have no resolution to determine the response of plants and other related microbes in response to mixed hydrocarbons (Sharma et al., 2023). Multi-omics integration (genomics, transcriptomics, proteomics and metabolomics) offers a system-level interpretation of such interactions, but results in big, complex data that is difficult to understand (Chen et al., 2023).

## 7 Challenges and future directions

Even though machine learning use in bio-augmentation and phytoremediation are gaining success, some important challenges remain that impede widespread application. One major problem is datasets availability and quality. Some machine learning (ML) models require that training datasets be large, well-organized and consistent, but much environmental data, particularly from contaminated sites, is sparse and inconsistent (Alghamdi et al., 2025).

Combining different types of biological data like genes, RNAs, and metabolites with environmental data is tough (Goodrich et al., 2024). Stating a correct prediction alone may not satisfy consumers. They need to understand why the model is making that call or decision as this will inform consumers if the model is transparent or not. Such clarity will show why a certain plant-microbe combination will perform well under specified conditions or not (Madanchian, 2024).

This research contributes meaningfully to respiratory disease understanding and prevention by addressing the environmental determinants of pulmonary health. Hydrocarbon pollutants particularly PAHs are respiratory toxicants that induce chronic inflammation, oxidative stress, and carcinogenesis within pulmonary tissues. By developing ML driven frameworks for optimizing bioaugmentation and phytoremediation, this study indirectly supports respiratory health through environmental detoxification and risk reduction (Alqahtani J. S. et al., 2025; Alasmari et al., 2025; Alqahtani M. M. et al., 2025). The integration of multi-omics data enhances predictive accuracy in identifying microbial and plant systems capable of degrading airborne and soil hydrocarbons, ultimately lowering population exposure to carcinogenic and respiratory irritants. Such advancements not only align with environmental health protection goals but also provide a translational pathway for mitigating respiratory disease incidence linked to industrial pollution. This intersection between environmental biotechnology and respiratory care epidemiology highlights the necessity of sustainable remediation as a proactive public-health intervention (Alghamdi et al., 2021).

### 7.1 Machine learning optimism in phytoremediation and bioaugmentation

The optimism surrounding the integration of machine learning (ML) into hydrocarbon bioremediation does not reflect the actual real-world variability and context-dependence of ML outcomes. Successes reported in the literature are predominantly from controlled laboratory scenarios, while the field (temper, moisture, and nutrient levels) conditions impose the greatest uncertainties to ML performance (Trivedi et al., 2025; Martins et al., 2022).

The literature on machine learning (ML) reported the Random Forest (RF), Support Vector Machine (SVM), Artificial Neural Network (ANN), and Deep Learning (DL) tools giving excellent performance (*R*^2^ > 0.85 or accuracy >80%) predictions, while transferring the ML from laboratory conditions to the field presented the greatest uncertainties in the overall ML performance (Saidi et al., 2025). Uncertainties associated with soil moisture, temperature, nutrients, and microbes placed severe prediction capabilities of the ML, causing increases of 20–30% in whole predictions of the machine learning transfer to the soil environment (Saidi et al., 2025).

In the absence of sufficient validation benchmarks, and the overall absence of reporting negative results, a severe publication bias leads to a form of over-optimism in the field, while gaining publicity, and even monetary incentives, for failing models (Saidi et al., 2025). ML models typically have excellent intra-model validation, while failing in actual field environments due to the over-optimism of the modeled results (framed in the absence of negative outcomes). Overfitting results from the large number of features, negating the effectiveness of the models, and leading to even greater levels of prediction failure of the models (Aliferis and Simon, 2024).

Table 3 provides a summary of typical applications of machine learning in hydrocarbon bioaugmentation and phytoremediation, including some performance metrics and known limitations and modes of failure. The comparison demonstrates that while machine learning may be able to surpass traditional kinetic or regression-based models in pattern recognition and non-linear forecasting, this advantage is much less pronounced when the data in terms of quality, quantity, or representativeness is lacking.

**Table 3:** Performance metrics of representative ML applications in hydrocarbon bioaugmentation, phytoremediation, and the related documented constraints and failure modes.

Context of study	ML algorithm	Type of data	Performance	Validation method	Key failure mode(s)	References
Lab-scale degradation of PAHs	Random forest	Soil chemistry and genomics	R2 is nearly equal to 0.90	k-fold CV	Overfitting due to small sample size	Chala and Ray (2023)
Mesocosm bioremediation	ANN	Enzyme activity and metagenomics	Accuracy is approx. 85%	Train test split	Poor generalization to new soil types	Galanova et al. (2025)
Field scale soil spill oil	SVM	Physicochemical and meta-transcriptomics	RMSE increases by ~25% in the field	External validation	Data sparsity, temporal drift	Cao et al. (2022)
Phytoremediation trials	Deep learning	Meta-genomics and plant traits	F1 score is approximately 0.78	Limited external testing	High computational cost; issues of interpretability	Toukak et al. (2026)

CV, Cross Validation.

In summary, while future studies will be required to advance the current state of proof-of-concept models, it will also be important to consider the reporting of uncertainty, adopt more standardized validation processes and consider the use of hybrid models that combine a more mechanistic approach with a data driven approach.

The power of machine learning (ML) in optimizing bioaugmentation and phytoremediation is considerable. While soil and hydrocarbons are likely the areas where the application of ML is the most constrained in regard to methodological, biological, and socio-technical constraints, there are several issues that will need to be critically addressed to avoid overgeneralization of the issues (Zhang et al., 2024).

### 7.2 Algorithmic bias and representation gaps in omics-driven models

The most important issue, in the case of ML models trained with multi-omics data, is the lack of sufficient data to help create ML models because of the biased data coverage in the genomic and metagenomic public information due to the unequal representation of certain functional genes and microbial taxa. More specifically, the publicly available metagenomic and genomic datasets still suffer from a lack of diversity in the soil-dwelling microbial taxa primarily due to the focus on model organisms that have been previously studied, and as a result, the microbial taxa that are present in soil that is tropical, arid, or chronically contaminated (Wang et al., 2023; Flores et al., 2023). Consequently, ML models are likely to be biased by learning patterns that belong to the dominant taxa, and therefore, they are likely to misestimate the degradation potential of a given community due to the presence of a dominant yet uncultured or rare microorganism.

The lack of a key community member in hydrocarbon remediation will have an ill-defined or low-abundance role, resulting in the ML-derived model failing to provide a representative answer that appears to be correct. The ML model will provide seemingly accurate predictions based on data that is biased, and the model will likely fail to perform in areas where the soil or microbial community is different (Zhang et al., 2023). The solution to this problem includes a need for more reference genomes, bias-aware learning algorithms, and the use of ecological datasets as a source of information in ML frameworks.

### 7.3 Even on heterogeneous and dynamic soils: performance limitations

With regards to evidence that ML has outperformed most classical statistical approaches for specific use cases in a controlled laboratory setting, there are still numerous studies that evidence reduced performance or even failure in heterogeneous field environments.

Though Adeshina et al. (2020) described encountering a high (>20\%) false positive rate when extrapolating from their training domain on environmental datasets for ML classifiers, especially for highly spatially heterogeneous systems, Jumbo et al. (2022) also describes ML models that predicted petroleum hydrocarbon degradation in mesocosms, but in field soils where moisture and nutrient fluctuations disrupted learned patterns, the models greatly underestimated the biodegradation potential.

In some phytoremediation studies where data availability was limited, process-based kinetic models sometimes matched or outperformed ML approaches (Sharma et al., 2023). For example, Sharma et al. (2023) described ANN -based predictions of PAH removal, where models and mechanistic models were able to provide field scale estimates, whereas models suffered from overfitting and high dimensional input and small sample sizes. These examples provide evidence that when attempting to utilize sparse, or noisy datasets, the real world complexity of the datasets, the advantages of ML diminish.

### 7.4 Scalability and equity challenges in low-resource settings

The most notable shortcoming of ML-enabled bioremediation in low-resource contexts is that it is not scalable. Low-resource communities have limited access to tools such as high-throughput sequencing, computational resources, and advanced analytics. The generation of multi-omics data is expensive. Additionally, most machine-learning (ML) processes depend on the use of cloud services, or specialized hardware which, is also not available in areas most impacted economically and environmentally by hydrocarbon pollution (Vuković Domanovac et al., 2025).

Furthermore, reliance on data-intensive practices may reinforce the widening of the gap between technologically advanced, highly funded research institutions and the most disadvantaged communities in need of remediation. In the absence of simplified, transferable frameworks, low-cost proxy indicators, ML- driven remediation is unlikely to be implemented beyond laboratory and pilot-scale demonstrations.

### 7.5 Incorporation of abiotic variables in machine learning remediation models

There is an increase in the application of machine learning (ML) in the fields of bioaugmentation and phytoremediation, but still a large gap in how the contexts of the environments are considered across models. Although the latest studies are incorporating multi-omic datasets (metagenomic, transcriptomic, and metabolomic), many of the top performing machine learning models include nonliving (abiotic) environmental factors, including pH, salinity, temperature, oxygen, the amounts of nutrients, moisture, and other soil and water variables, as they are considered to be primary drivers of microbial activity and the rate of hydrocarbon degradation. Models incorporating biological factors from omics with abiotic factors are more reliably predictive and have greater relevance in the field, especially in heterogeneous or dynamic soil settings.

Conversely, a small number of studies rely on using only omics data for ML models, which are often created in controlled laboratory or mesocosm conditions with deliberately minimized environmental variation. Although these studies report a high level of accuracy on regressions and classifications, their application in the field is minimal, as they ignore the fluctuating abiotic drivers which results in a less robust and generalizable model across multiple site. These examples demonstrate a gap in more complex machine learning models that include the biological and nonliving environmental variables and a lack of clear and consistent reporting of the input variables, which constrains the predictive power and utility of ML-based remediation in actual environments.

### 7.6 Regulatory and biosafety considerations in ML-guided bioaugmentation

Considering the use of ML bioaugmentation strategies in practice raises new regulatory and biosafety challenges that are currently under-represented in literature. Most ML frameworks perform predictive optimization of microbial consortia or functions while disregarding the positive/negative regulatory constraints applicable to the release of organisms into the environment, especially the engineered or modified consortia, or organisms of foreign origin. Most jurisdictions have laws that apply to the release of engineered organisms and require reviews that examine potential microbe persistence, gene transfer, disruption of microbial communities and ecosystems, and opportunistic pathogenesis. These issues are not offered as constraints in regulatory frameworks and are rarely included in the biosafety frameworks for ML microbial optimization in the pathogen control process.

Considering biosafety issues, ML bioaugmentation strategies may lead to the selection of less benign potential degraders whose ecological or health-related impact may be adverse, especially when datasets fail to capture long-term conditions and outcomes in the field. Some ML models are less transparent than others, and this raises issues in the approval process due to the small complexity and interpretability, which impacts traceability, accountability, and compliance with the precautionary principle. In low-resource countries, where there may be little or no resource regulatory controls, vision post-release monitoring and adaptive risk control, these issues are further pronounced.

ML-enabled future bioaugmentation frameworks should, as a design choice, include compliance with regulatory, biosafety, incorporation of exclusion of high-risk taxa, ecological containment, and hybrid biosafety/biotechnology frameworks. Safeguards integrated into ML workflows will be necessary to ensure data-driven remediation strategies are effective, responsible, and acceptable.

### 7.7 Future directions

For future research to be more robust and globally applicable, it should focus on the following:

- A bias- and underrreported- taxa- and annotation- agnostic ML frameworks that are interpretable (Jui and Rivas, 2024);
- The use of hybrid models that integrate the mechanistic and the data-driven and predictive to improve extrapolation in data-poor environments (Sansana et al., 2024);
- Standardized frameworks that include the reporting of positive and negative error rates, uncertainty, and where the models fail (Simmonds et al., 2022);
- The use of scalable and resource sensitive features, such as ML- models that utilize a reduced number of features and sensor- driven proxies to facilitate low-resource country adoption (Zhang et al., 2026).

“Multiple heterogeneous data types” effectiveness is dependent on data quality and context. The translation of ML innovation into dependable, globally relevant remediation strategies, will require a cautious, transparent, and equity-oriented approach.

Future efforts should aim to create high-resolution, open-access databases for diverse soil types and contaminants, hybridize the mechanistic (process-based) model with an ML model for better veracity and interpretability, and enhance edge computing and real-time analytics for feedback-oriented adaptive bioremediation.

## 8 Conclusion

The integration of machine learning (ML) into bioaugmentation and phytoremediation represents a significant advancement in the management of hydrocarbon-contaminated soils. By enabling predictive modeling, real-time environmental optimization, and precise plant-microbe selection, ML addresses key limitations of traditional remediation methods such as slow uptake rates, site variability, and empirical trial dependence. Case studies have demonstrated the effectiveness of ML in improving contaminant degradation efficiency by 60% (Janga et al., 2023), streamlining species selection by 25% in terms of accuracy (Blessing and Olateru, 2025), and supporting adaptive, site-specific strategies that reduce remediation time by 35% in variable soil conditions (Janga et al., 2023).

Despite challenges such as data scarcity, model interpretability, and integration of complex multi-omics datasets, ongoing advances in computational tools, sensor technology, and environmental data collection are paving the way for more intelligent and automated remediation systems. Future developments should prioritize standardized data frameworks, hybrid modeling approaches, and accessible decision-support tools to ensure broader application in field settings.

The table shows ML application in integration with various omics layers in microbial consortia studies and their main limitations (Table 4).

**Table 4:** Summary of synergies in ML integration with omics in hydrocarbon bioremediation.

Omics layer	ML contribution	Key synergy	Application	Main limitation
Genomics	Feature selection, gene-function prediction	Connects catabolic genes to degradation potential	Identification of hydrocarbon-degrading pathways	Bias toward well-annotated model organisms
Transcriptomics	Pattern recognition, temporal modeling	Connects gene expression dynamics to stress response and activity	Monitoring microbial response during remediation	High noise; context-dependent expression
Proteomics	Classification, multivariate integration	Bridges genotype–phenotype gap via enzyme-level insights	Prediction of active degradation enzymes	Limited coverage; technical variability
Metabolomics	Regression, clustering	Associates metabolic fingerprints with degradation rates	Tracking hydrocarbon breakdown products	Small datasets; metabolite ambiguity
Microbiomics	Community-level modeling, network inference	Links community structure to functional outcomes	Predicting consortium performance	Reduced transferability across sites
